# A Novel Strategy to Simplify the Procedures in Treating Complicated Coronary Bifurcation Lesions: From a Bench Test to Clinical Application

**DOI:** 10.3389/fcvm.2022.854063

**Published:** 2022-04-01

**Authors:** Dongdong Li, Wenshuai Ma, Pengyun Liu, Hao Liu, Baobao Bai, Mingming Zhang, Wangang Guo

**Affiliations:** Department of Cardiology, Tangdu Hospital, Air Force Medical University, Xi'an, China

**Keywords:** bifurcation, bench test, jailed balloon technique, proximal optimization technique, provisional stenting

## Abstract

**Background:**

Although provisional stenting strategy based on jailed balloon side branch (SB) protection could be useful for high-risk bifurcation lesion in certain clinical scenarios, its complexity still gives rise to procedure complications. We proposed a novel strategy, the jailed balloon proximal optimization technique (JB-POT), to simplify the procedures in treating complex coronary bifurcation lesions (CBLs). The present study was designed to verify the safety and efficacy of JB-POT under bench testing and clinical circumstances.

**Methods:**

After a stent was deployed in main vessel (MV) with a balloon jailed in SB, POT and post-dilation of the stent were performed without retrieving the jailed balloon. A re-POT was performed 2 mm away from SB branching point to minimize proximal stent malapposition. The JB-POT procedure was performed on 10 samples of a silicone bifurcation bench model, and optical coherence tomography (OCT) was utilized to evaluate stent deployment. From December 2018 to July 2021, a total of 28 consecutive patients with true CBLs treated with JB-POT were enrolled. Immediate procedure results were observed, and clinical follow-ups were performed.

**Results:**

The bench test showed that JB-POT did not induce significant stent malapposition, underexpansion or distortion, as indexed by the malapposition rate, minimum stent area (MSA), eccentricity index and symmetry index determined through OCT. Under clinical circumstances, JB-POT did not induce significant malapposition, underexpansion or distortion. Among the 30 lesions, there was no primary endpoint event defined as SB occlusion, need to rewire the SB with a polymer-covered guide wire, or failure to retrieve a jailed wire or balloon. One rewiring event and 0 double stenting events occurred as secondary endpoint events. One patient died of heart failure in the 8th month after discharge.

**Conclusions:**

The JB-POT protocol, which tremendously simplifies the current standard provisional stenting procedure in complicated bifurcation lesions, shows acceptability in safety and efficacy. Hence, it might become an applicable strategy for treating high-risk bifurcation lesions, especially those with multiple risked SBs.

## Introduction

Coronary bifurcation lesions (CBLs) are involved in 15–20% of all percutaneous coronary interventions (1). A provisional stenting strategy based on jailed balloon side branch (SB) protection could be useful for high-risk bifurcation lesions in certain clinical scenarios according to the recent studies (2, 3). In the traditional jailed balloon-based provisional stenting (JB-PS) protocol, SB rewiring, stent dilation after deployment (post-dilation), proximal optimization technique (POT), SB dilation (if necessary), final kissing and repeated POT are frequently required. Although POT was proven to optimize proximal main vessel (MV) stent apposition and SB opening in some bench studies (4, 5), further research showed that POT caused 6–10% SB strut jailing in bench studies and 30% SB FFR < 0.75 in clinical trials (6, 7). Although the jailed balloon technique (JBT), including both conventional and modified JBT (8), prevents carina and plaque shifts during stent deployment, POT could still cause SB narrowing by the carina or plaque shift mechanism after the jailed balloon has been retrieved when POT is not accurately positioned. As a result, rewiring SB and SB dilation become routine manipulations before POT and post-dilation. However, these manipulations are often time-consuming and troublesome, especially when they need to be repeated to protect each SB from occlusion in diffuse MV bifurcation lesions accompanied by multiple endangered SBs.

Hereby, we simplified the provisional stenting strategy from JB-PS to the jailed balloon proximal optimization technique (JB-POT) by performing post-dilation and POT with a balloon jailed in the SB. This strategy limits carina/plaque shift during post-dilation and POT, and it saves the steps of SB rewiring, SB dilation and final kissing, which would substantially shorten the procedure time and reduce the complexity of the CBL intervention under certain circumstances. Our concern about this strategy is whether it would induce unacceptable malapposition, underexpansion and/or distortion and whether it would effectively protect the SB from occlusion. The present study utilized a silicosis CBL model to test the proximal stent apposition, proximal stent expansion and proximal stent formation. A retrospective observation was performed to examine the efficacy and safety of JB-POT in real-world applications.

## Materials and Methods

### JB-POT Rationale and Protocol

**Figure 1:**
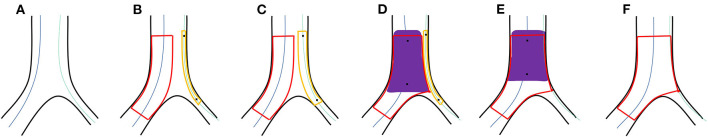
**(A)** Both the MV and SB are wired. **(B)** The MV stent is then deployed with a balloon jailed in the SB. **(C)** The jailed balloon is dilated at 6–8 atm if SB blood flow is degraded. **(D)** POT and post-dilation of the distal stent were performed with non-compliant balloons of corresponding sizes. **(E)** The jailed balloon is retrieved and rePOT is performed 2 mm away from the SB branching point. **(F)** Final effects are examined.

In CBLs of higher angiographic complexity, the amount of atherosclerotic plaque located in the polygon of confluence is higher. Stenting the MV is known to have the potential to compromise the SB due to the occurrence of plaque and carina shifts. JBT is an effective strategy to prevent plaque and carina shifts (3, 9, 10). In the standard JB-PS protocol, the jailed balloon is retrieved after stent deployment and cannot protect the SB from carina/plaque shift and narrowing during POT and post-dilation. Hence, rewiring the SB, dilating the SB ostium and final kissing are often required to avoid SB complications. As shown in Figure 1, the JB-POT technique was designed to avoid SB rewiring and final kissing to simplify the provisional stenting protocol. After jailed balloon MV stenting, a non-compliant balloon of the proximal MV diameter was used to perform POT with the jailed balloon maintained. SB blood flow was then checked by angiography, and the SB jailed balloon was dilated if SB flow was degraded at the time. Furthermore, the jailed balloon was retrieved gently, and repeated POT (re-POT) was performed 2 mm away from the SB branching point (avoiding influencing SB ostium) to revise the probable malapposition or underexpansion. After initial jailed balloon POT, rewiring, side branch dilation and kissing would be performed if side branch thrombolysis in myocardial infarction (TIMI) flow degraded (<3), side branch residual stenosis was over 90% for non-left main (LM) lesions or over 75% for LM lesions, or dissection type over A (11, 12). As the previous trials showed, SB [except large left circumflex coronary artery (LCX)] residual stenosis was often not functionally meaningful. In side branch of small territory, it is only necessary to keep it (side branch) open (13).

### Bifurcation Phantom

**Figure 2:**
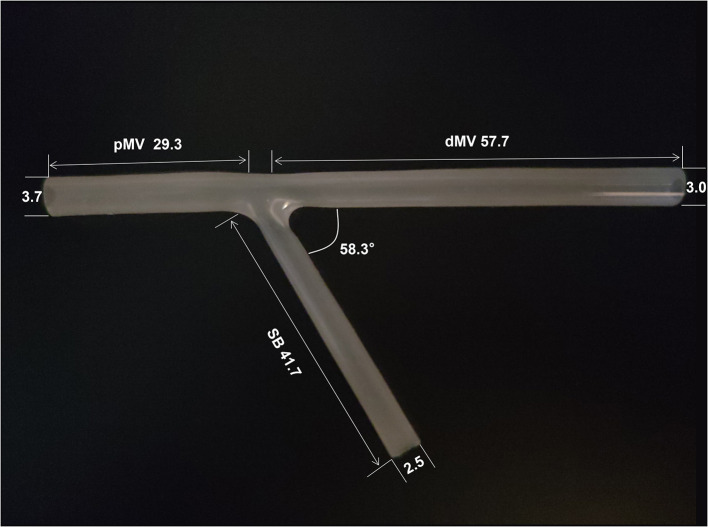
Bench test model of bifurcation stenting. The length of the pMV, dMV and SB is 29.3, 57.7 and 41.7 mm respectively. The inner lumen diameter of the pMV, dMV and SB is 3.7, 3.0 and 2.5 mm respectively. The bifurcation angle is 58.3°. pMW, proximal main vessel; dMV, distal main vessel; SB, side branch.

3D printing semitransparent silicone models of the coronary bifurcation artery were made by Ningbo Trando 3D Medical Technology Co., Ltd. Their inside diameters were 3.7 mm for proximal MV (D_pMV_), 3.0 mm for distal MV (D_dMV_), and 2.5 mm for SB (D_SB_), complying with Finet's law (D_pMV_ = 0.678 [D_dMV_ + D_SB_]) (Figure 2) (14, 15). The silicone material had similar elastic characteristics according to the EBC bench test consensus (16). The semitransparent characteristic was appropriate for testing or observing the simulated intervention procedures.

### Patients

**Figure 3:**
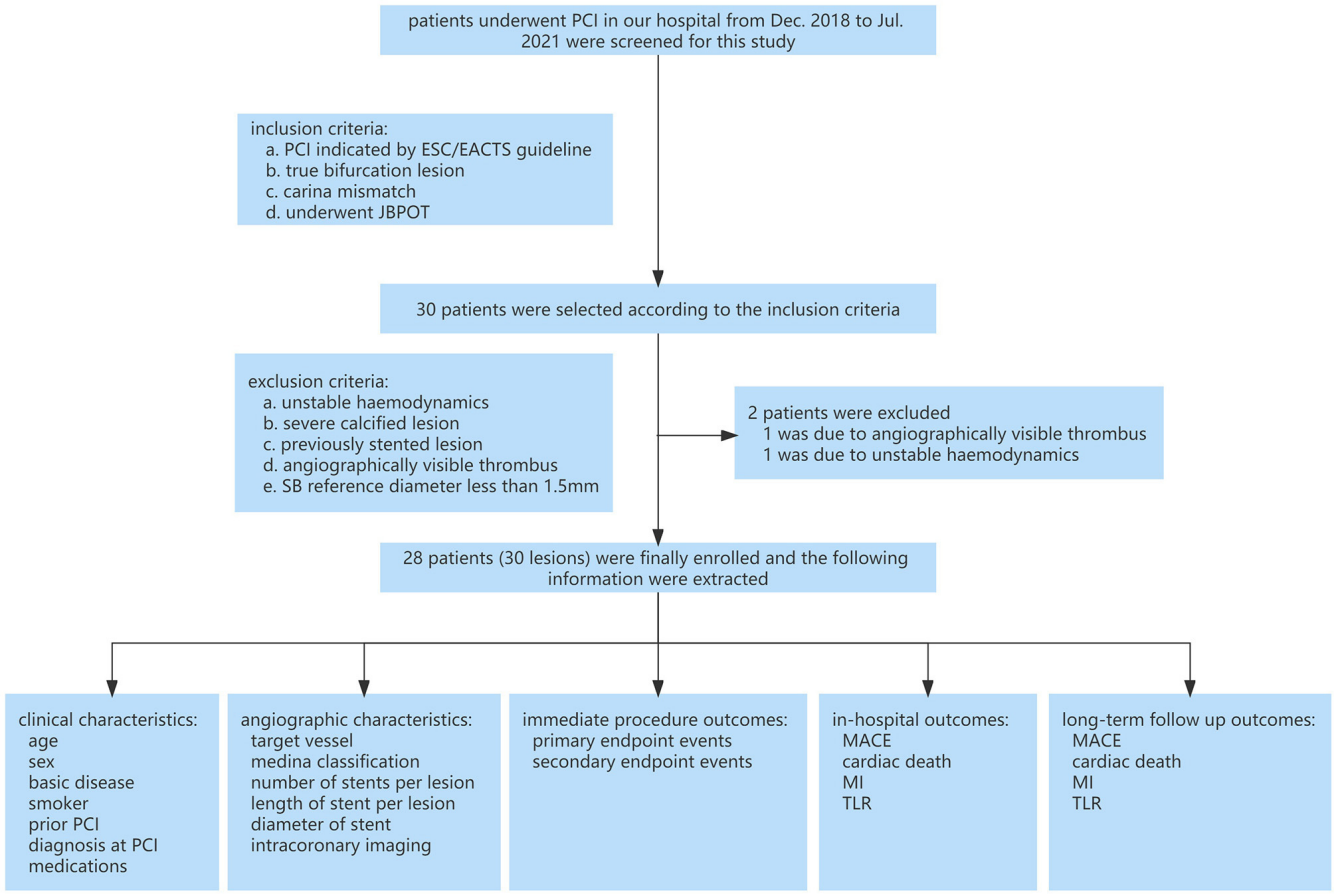
Clinical study flowchart. PCI, percutaneous coronary intervention; ECA, European Society Of Cardiology; EACTS, European Association For Cardio Thoracic Surgery; JB-POT, balloon-jailed proximal optimization technique; MACE, major adverse cardiovascular events; TLR, target lesion revascularization; MI, myocardial infarction.

In our hospital, 30 patients underwent JB-POT-based provisional stenting from December 2018 to July 2021. Twenty eight patients were enrolled in this study by a retrospective analysis. One patient was excluded due to angiographically visible thrombus, and another was excluded due to unstable hemodynamics (Figure 3). The indications for PCI included stable coronary disease and acute coronary syndrome, which were further determined by the results of preprocedural exercise electrocardiography, quantitative coronary angiography (QCA) or intravascular imaging. In this study, bifurcation lesions with risked SBs and significant MV stenosis as indicated by the 2018 European Society of Cardiology/European Association for Cardiothoracic Surgery guidelines on myocardial revascularization were selected (17). Risked SB was defined as true bifurcation lesions (Medina 1,1,1; 0,1,1; or 1,0,1) complying with carina mismatch according to the Vassilev theory (18), which referred to carina length equal to or longer than ostial residual width determined by QCA.

The exclusion criteria were as follows: (a) unstable hemodynamics; (b) severe calcified lesions in the CBL (significant haziness in coronary angiography, ≥90° or over 300 μm thick in intravascular ultrasound or OCT images); (c) previously stented lesion; (d) angiographically visible thrombus; and (e) lesions in which the SB reference diameter was <1.5 mm.

### Diagnostic and Therapeutic Procedure

Coronary angiography was performed in all patients after intracoronary administration of nitroglycerin (0.5 mg). The SB ostial segment was evaluated using two orthogonal coronary angiographic images, showing the SB ostium as clearly as possible. The baseline QCA was performed with images obtained before stent implantation. All lesions were treated using the JB-POT-based provisional stenting technique. Post-dilatation of the distal MV stent was performed by choosing a non-compliant balloon according to the distal MV diameter, and POT was performed by choosing a balloon according to the proximal MV diameter. The two orthogonal angiographies selected in the diagnostic phase were repeated after stent implantation to evaluate the SB ostial region.

### Intracoronary Imaging Measurement

OCT or intravascular ultrasound (IVUS) was performed based on the operators' criteria before, during and after the PCI procedure. OCT imaging was obtained using a Dragonfly™ Duo OCT imaging catheter (LightLab™ Imaging, Inc., Westford, MA, USA) and C7™XR system/OPTIS™ system (C7™XR system, LightLab™ Imaging, Inc., Westford, MA, USA; OPTIS™ system, Abbott Medical, Westford, MA, USA). Briefly, the OCT imaging catheter was advanced distal to the MV target lesion over a 0.014-inch conventional angioplasty guide wire through a 6 or 7 Fr guiding catheter. Pullbacks were performed during a continuous injection of 3.5–4 ml/s contrast media through the guiding catheter to remove blood from the field of view after the intracoronary administration of 0.5 mg nitroglycerin. Images were acquired at 100 fps/180 fps and an automated pullback speed of 20 mm/s. The IVUS catheter was advanced at least to the distal (> 10 mm) of lesion or stent edge after intracoronary administration of nitroglycerin. Automated pullback was set at 0.5 mm/s on a commercially available imaging system with a 40-MHz mechanical transducer (Boston Scientific Corporation, Natick, MA, USA).

**Figure 4:**
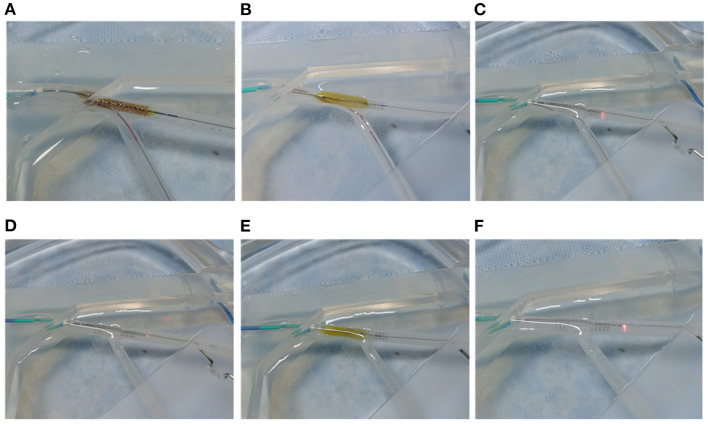
JB-POT bench test procedures. **(A)** Perform MV stenting with a balloon jailed in the SB. **(B)** Perform POT pullback at the carina level with the jailed balloon maintained. **(C)** Retrieve the jailed balloon and perform OCT evaluation with the jailed wire remaining. **(D)** Perform OCT pullback after retrieving the jailed wire (experimental arm). **(E)** Perform POT at the carina level to correct the potentially JB-POT-induced stent distortion. **(F)** Perform the final OCT run (self-control arm).

For the bench test, three OCT pullbacks were acquired after retrieving the jailed balloon, retrieving the jailed wire and performing the final POT respectively (Figure 4). Longitudinal sections and cross-sections were analyzed. The malapposition rate was calculated as the percentage of the total number of malapposed struts divided by the total number of proximal struts (SB ostium struts not included) (19). The minimum stent area (MSA) was defined as the minimum stent area of the frames within the proximal MV. The eccentricity index was computed as the ratio of the maximum to minimum scaffold diameter (8); the symmetry index was defined as the maximum scaffold diameter divided by the minimum scaffold diameter in the minimum frame (7, 20). Briefly, malapposition was defined as a lack of contact of at least 1 strut with the underlying vessel wall (at least 300 μm, in the absence of a side branch) with evidence of blood flow behind the strut according to the expert consensus document of the European Association of Percutaneous Cardiovascular Interventions on intravascular imaging (20).

### Endpoint Events

Primary endpoint events were defined as SB TIMI grade 0–1, SB rewiring failure, a need to rewire the SB by guide wire with polymer tip and sleeve, failure to retrieve SB jailed balloon and/or wire, malapposition distance ≥ 300 μm (SB ostium not included), minimal stent area (MSA) ≤ 5.5 mm^2^ for left main or ≤ 4.5 mm^2^ for non-LM lesions, and stent expansion rate ≤ 80%. The secondary endpoint events included SB TIMI grade 2, a need to rewire the SB by working-horse guide wire, final kissing, and/or rescue double stenting as decided by operators (21).

### In-hospital and Long-Term Clinical Outcomes

In-hospital clinical outcomes were defined as major adverse cardiovascular events (MACE) including target lesion revascularization (TLR), myocardial infarction (MI) and cardiac death before discharge. All patients were followed up after discharge to observe the rate of MACEs as an indicator of long-term clinical outcomes (22).

### Statistical Analysis

Statistical analyses were performed using SPSS 26.0. Paired *t* test was used to analyze the bench test data and *P*-value < 0.05 was considered significant.

## Results

### Bench Test

**Figure 5:**
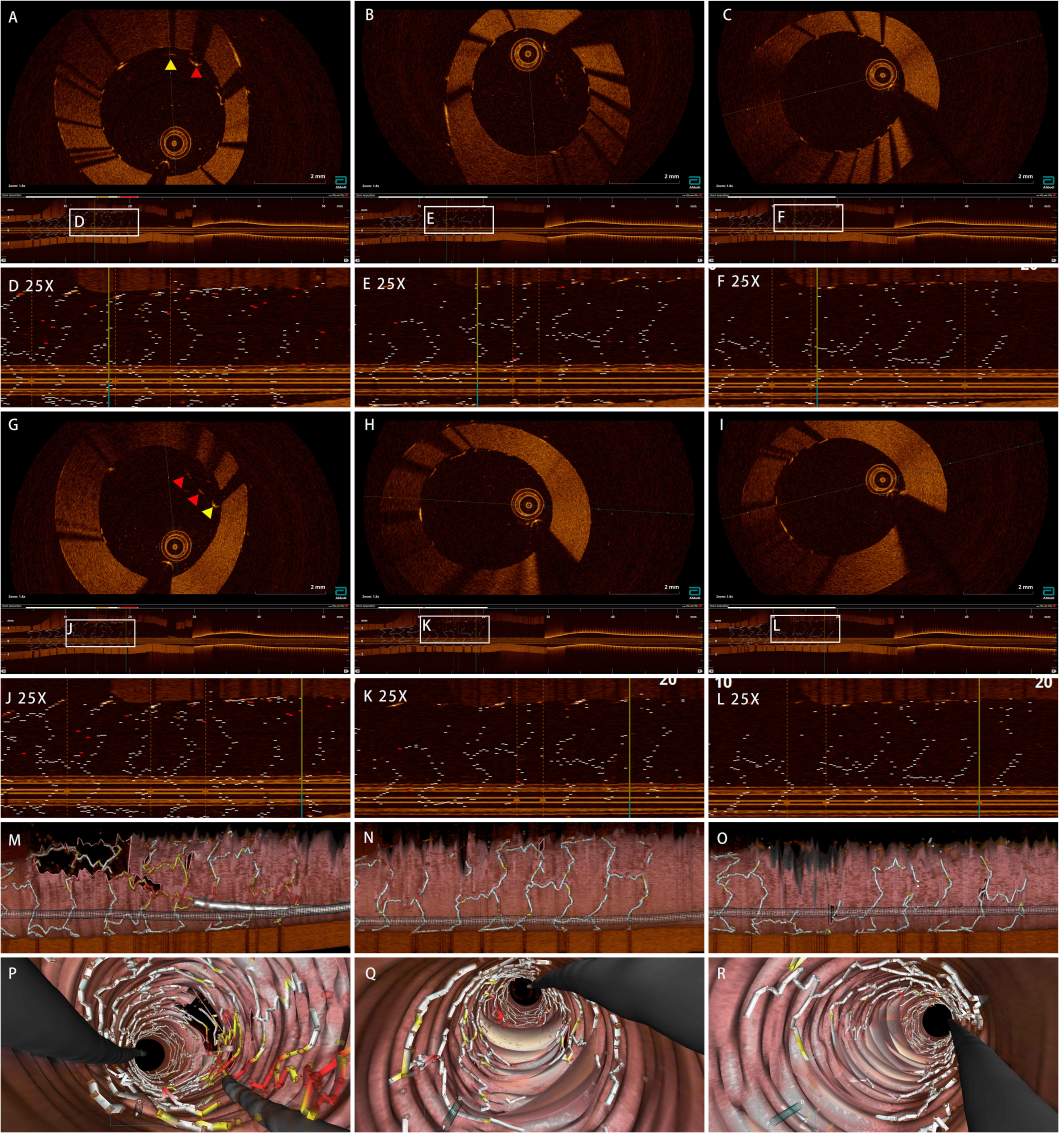
Effect of JB-POT procedures on stent deployment examined by OCT. **(A,G)** Cross-sections of JB-POT run before jailed wire retrieved. **(B,H)** Cross-sections of JB-POT final run. **(C,I)** Cross-sections of self-control run. **(D,J)** Longitudes of JB-POT run before jailed wire retrieved. **(E,K)** Longitudes of JB-POT final run. **(F,L)** Longitudes of self-control run. **(M,P)** 3D images of JB-POT run before jailed wire retrieved. **(N,Q)** 3D Images of JB-POT final run. **(O,R)** 3D Images of self-control run. Red arrows, malapposition distance ≥300 μm; yellow arrows, 200 μm ≤Malapposition distance <300 μm. JB-POT, jailed balloon proximal optimization technique; OCT, optical coherence tomography.

**Figure 6:**
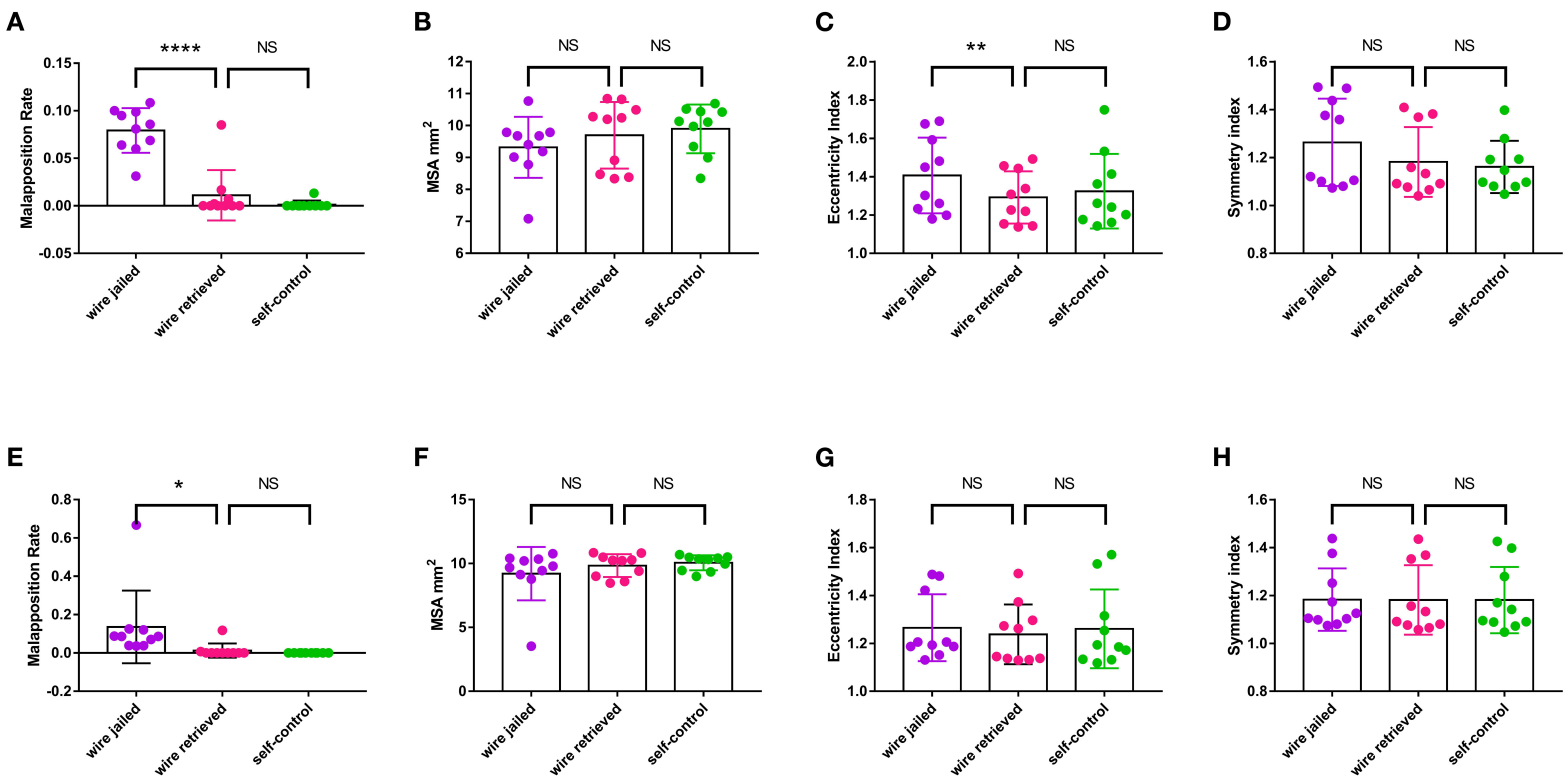
Effect of JB-POT procedures on stent deployment parameters determined by OCT. **(A–D)** Malapposition rate, MSA, eccentricity and symmetry of the whole stent. **(E–H)** Malapposition rate, MSA, eccentricity and symmetry of the 2 mm stent segment adjacent to SB take-off. MSA, minimum stent area. NS, no significant. *p < 0.1. **p < 0.05, ****p < 0.0001. *n* = 10.

Ten bench tests were conducted as shown in Figure 4. After the JB-POT procedure, OCT was used to determine the parameters of stent deployment, which were taken as the experimental arm. The non-compliant balloon for POT was reused to fully dilate the proximal stent following jailed balloon and wire retrieval, and the corresponding stent deployment status was regarded as self-control. Typical OCT images are shown in Figure 5. After retrieval of the jailed balloon and wire, the gap between the stent and vessel during the JB-POT maneuver vanished. The stent apposition, expansion and formation between the two arms were compared. Malapposition rates were 0.011 ± 0.027 in the experimental arm and 0.001±0.004 in the self-control arm. The MSAs were 9.687 ± 1.043 and 9.896 ± 0.761 mm^2^ for the experimental and control arms, respectively. The eccentricity indices were 1.292 ± 0.136 and 1.324 ± 0.194 for the experimental and control arms, respectively. The symmetry indices were 1.181 ± 0.146 and 1.161 ± 0.109 for the experimental and control arms, respectively. There were no statistically significant differences between the 2 arms regarding malapposition rates, MSAs, eccentricity indices or symmetry indices. However, a distanced re-POT was designed to be positioned 2 mm proximal to the SB branching point to avoid closing the SB ostium. Hence, the 2 mm segment apposition was not fixable in the present JB-POT protocol. There were no significant differences between the 2 arms regarding malapposition rates, MSAs, eccentricity rates or symmetry indices (Figure 6).

### Patients' Clinical and Angiographic Characteristics

The patients' clinical characteristics are summarized separately in Table 1 in terms of SB complications. Most patients were men with hypertension and dyslipidemia and were smokers, and all of them were diagnosed with acute coronary syndrome. According to our experience and previous studies (23–25), these patients are vulnerable to SB complications during one-stent crossover intervention. The angiographic characteristics are listed in Table 2. Most bifurcation lesions were located at the left anterior descending (LAD) coronary artery and classified into the Medina (1,1,1). In the present study, all of the bifurcation lesions had longer carina lengths complying with carina length mismatch. These characteristics indicate that the present study included bifurcation lesions with SBs mostly vulnerable to closure during the one-stent crossover strategy.

**Table 1:** Patient characteristics.

**Characteristics**	***n* = 28 patients**
Age, years	58.7 ± 12.0
Male, n (%)	19 (67.9%)
Hypertension, n (%)	18 (64.3%)
Diabetes mellitus, n (%)	7 (25.0%)
Dyslipidemia, n (%)	19 (67.9%)
Smoker, n (%)	14 (50.0%)
Prior PCI or CABG, n (%)	7 (25.0%)
Diagnosis at PCI, n (%)
STEMI	8 (28.6%)
NSTEMI	8 (28.6%)
UA	12 (42.9%)
Pharmaceutical medications, n (%)
Aspirin	28 (100%)
Clopidogrel	12 (42.9%)
Ticagrelor	16 (57.1%)
Beta blocker	17 (60.7%)
ACEI/ARB	13 (46.4%)
Statin	28 (100%)

*PCI, percutaneous coronary intervention; CABG, coronary artery bypass graft; STEMI, ST segment elevation myocardial infarction; NSTEMI, non-ST segment elevation myocardial infarction; UA, unstable angina; ACEI, angiotensin-converting enzyme inhibitors; ARB, angiotensin receptor blockers*.

**Table 2:** Angiographic and procedural characteristics.

**Characteristics**	***n* = 30 lesions**
Target vessel, n (%)
LAD-D1	17 (56.7%)
LAD-D2	9 (30%)
LCX-OM	3 (10%)
LM-LCX	1 (3.3%)
Medina classification, n (%)
(1,1,1)	20 (66.6%)
(1,0,1)	7 (23.3%)
(0,1,1)	3 (10%)
Number of stents per lesion, n	1 (100%)
Length of stent per lesion, mm	26.43 ± 5.83
Diameter of stent, mm	2.93 ± 0.45
IVUS	23 (76.7%)
OCT	7 (23.3%)
Procedure duration, min	50 ± 18
Consumption of contrast medium, ml	90 ± 25

*LAD, left anterior descending coronary artery; LCX, left circumflex coronary artery; LM, left main coronary artery; D1, the first diagonal branch; D2, the second diagonal branch; OM, obtuse marginal branch; IVUS, intravascular ultrasound; OCT, optical coherence tomography*.

### Application of JB-POT in Clinical Circumstances

JB-POT was performed in 30 bifurcation lesions of 28 patients from December 2018 to July 2021. There were no primary endpoint (JB-POT failure) events among these procedures, including SB TIMI grade 0–1, SB rewiring failure, a need to rewire the SB by guide wire with a polymer tip and sleeve, failure to retrieve the SB jailed balloon and/or wire, clinically relevant malapposition, MSA of ≤ 5.5 mm^2^ for LM or ≤ 4.5 mm^2^ for non-LM lesions, and stent expansion rate ≤ 80%. There was only one secondary endpoint event involving rewiring the SB by a working-horse guiding wire and final kissing (Table 3). There was no SB TIMI grade 2 occurrence, and rescue double stenting was performed. Specifically, MSA was 6.5 ± 2.6 mm^2^, the malapposition rate was 0, and the expansion rate (defined as MSA divided by distal stent area) was 85.4 ± 2.6% in the proximal stent segment. The mean amount of contrast medium was 90 ± 25 ml and the mean duration was 50 ± 18 min.

**Table 3:** Immediate postoperative endpoint events.

**Characteristics**	***n* = 30 lesion**
Primary endpoint events (JB-POT failure events)	
SB TIMI grade 0–1	0
SB rewiring failure	0
Needing to rewire SB by wire with polymer tip and sleeve	0
Re-wire SB with polymer-covered guide wire	0
Failure to retrieve SB jailed balloon and/or wire	0
Clinically relevant malapposition	0
Stent expansion rate ≤ 80%	0
MSA ≤ 5.5 mm^2^ for LM or ≤ 4.5 mm^2^ for non-LM lesions	0
Secondary endpoint events	
SB TIMI grade 2	0
Needing to rewire SB by working-horse guiding wire and final kissing	1 (3.3%)
Rescue double stenting	0

*JB-POT, jailed balloon proximal optimization technique; SB, side branch; MSA, minimal stent area; LM, left main; TIMI, thrombolysis in myocardial infarction*.

No MACE (including MI, TLR and cardiac death) occurred during the in-hospital period. There were 28 patients enrolled in this study, among whom two patients were lost in the follow up and the other 26 patients were contacted successfully for at least 6 months. The mean duration of follow up is 619 days. Of the 26 patients, 19 patients had reached 1 year follow-up. One patient died of heart failure in the 8th month and no patient suffered from MI or underwent TLR. In total, no patient suffered from MACE during the 6-month follow up and 1 (5.2%) patient suffered from cardiac death and MACE during the 1-year follow-up (Table 4).

**Table 4:** Clinical outcomes.

**Characteristics**	
Immediate clinical outcomes	*n* = 28 patients
MACE	0
Cardiac death	0
MI	0
TLR	0
Long-term clinical outcomes of 6 months	*n* = 26 patients
MACE	0
Cardiac death	0
MI	0
TLR	0
Long-term clinical outcomes of 1 year	*n* = 19 patients
MACE	1 (5.2%)
Cardiac death	1 (5.2%)
MI	0
TLR	0

*MACE, major adverse cardiovascular events; MI, myocardial infarction; TLR, target lesion revascularization*.

### Representative Case of JB-POT Application

**Figure 7:**
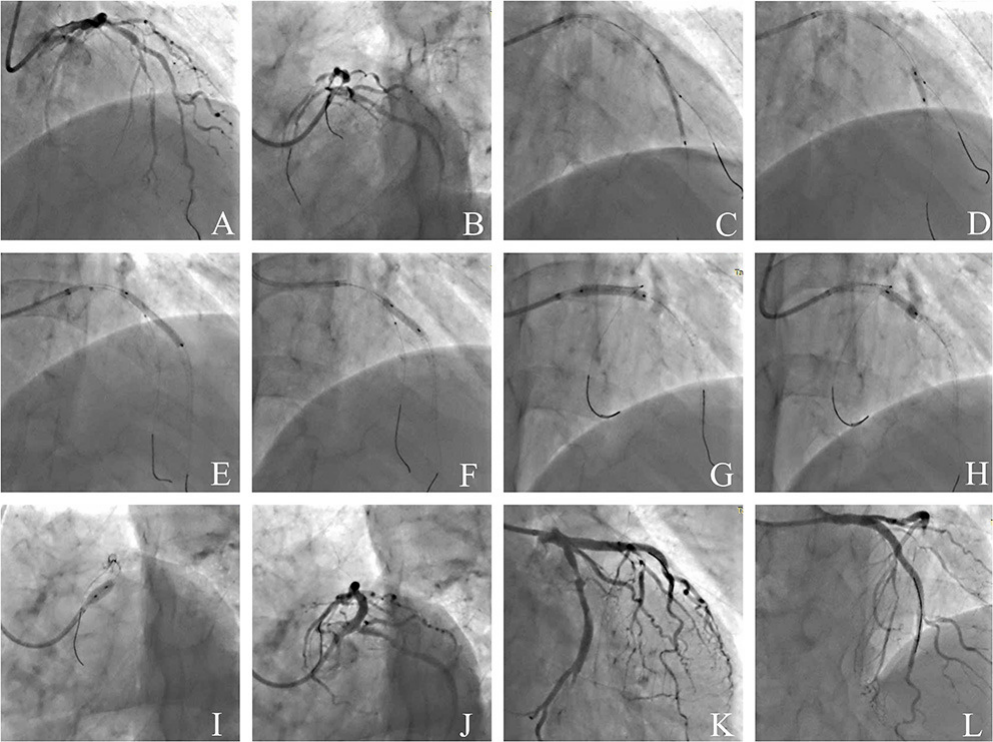
Coronary angiography illustrating the JB-POT in the intervention of a complicated bifurcation lesion. **(A,B)** Baseline angiography of diffused LAD-LM lesions with 3 true bifurcations (LAD-D1, LAD-S1, LAD-D2); **(C)** deploying a 2.5 × 29mm DES crossing D2 with a 1.5 × 15mm balloon jailed in D2; (D) post-dilation and JB-POT of the first stent with 2.5 × 12mm and 3.0 × 12 non-compliant balloons; **(E)** deploying a 3.0 × 29mm DES crossing D2 with a 1.5 × 15mm balloon jailed in D2; **(F)** JB-POT of the 3.0 stent with a 3.5 × 12 NC balloon, re-POT the 2.5mm stent and post-dilation of the 3.0mm stent with a 3.0 × 15 NC balloon; **(G)** deploying a 3.5 × 24mm DES crossing LCX with a wire jailed in LCX; **(H)** post-dilation of the 3.5mm stent and re-POT of the 3.0mm stent with a 3.5 × 12 NC balloon; **(I)** performing POT in LM with a 5.0 × 10 NC balloon; **(J–L)** final angiographic results. JB-POT, jailed balloon proximal optimization technique; LAD, left anterior descending coronary artery; LM, left main coronary artery; D1, the first diagonal -branch; D2, the second diagonal branch; S1, the first septal branch.

**Figure 8:**
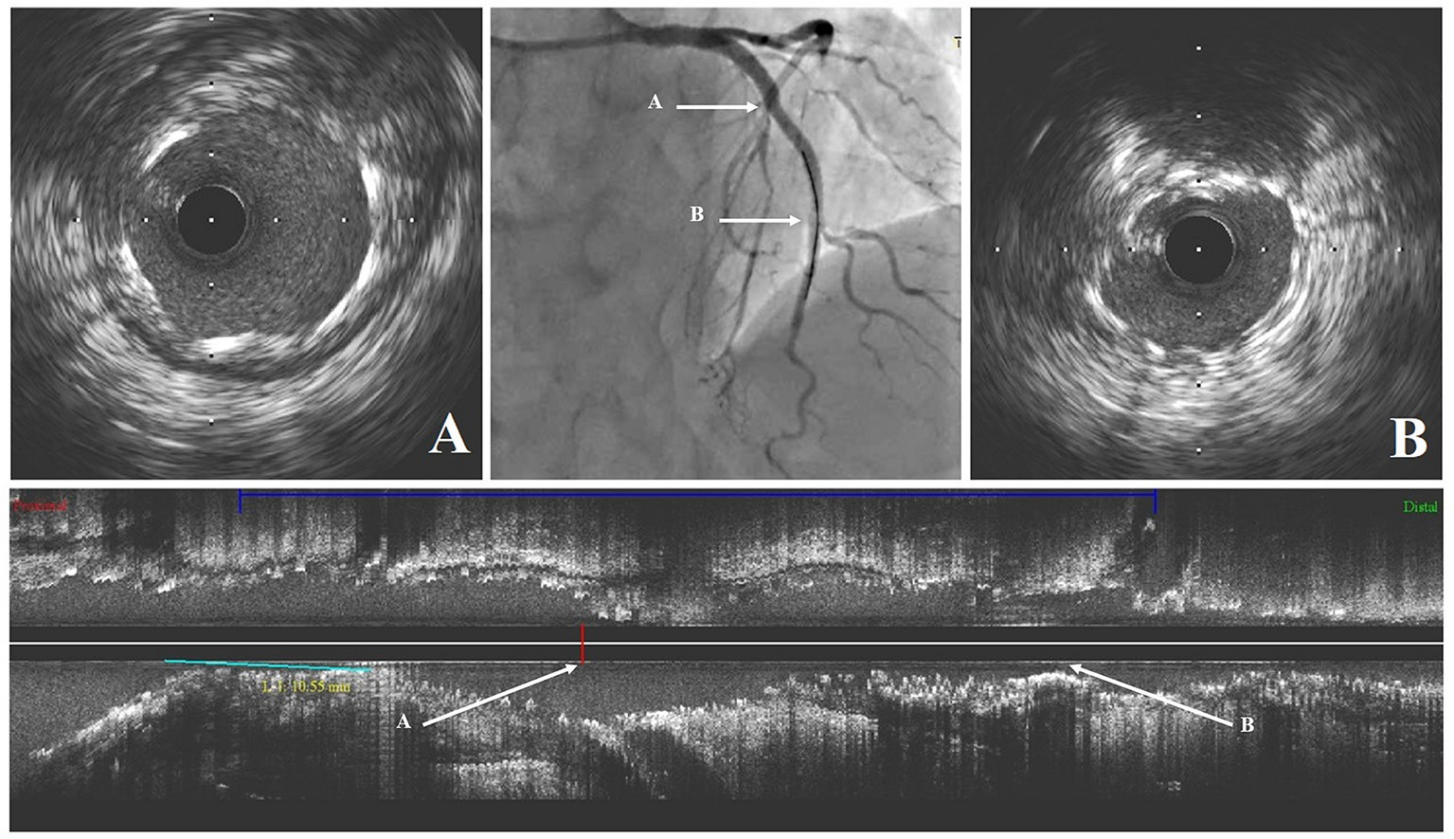
The intravascular ultrasound findings after stenting. **(A)** Cross-sectional intravascular image of LAD stent just proximal to the septal branch branching point; **(B)** Cross-sectional intravascular image of LAD stent just proximal to the 2nd diagonal branch branching point. LAD, left anterior descending coronary artery.

Figure 7 shows a representative case of JB-POT application in a coronary bifurcation lesion with risked SBs. Baseline coronary angiography LAD had diffused stenotic lesions from medium LAD to LAD ostium branching 2 diagonal branches and 1 septal branch. LAD-D1 was below 2 mm, and its territory was limited; hence, LAD-D1 did not need to be protected during the procedure. The other 2 bifurcation lesions were both Medina classifications (1,1,1). Baseline angiography showed both bifurcation lesions had a narrow carina angle and a short branching point-carina tip complying with carina length mismatch (18), which was demonstrated to be highly related to SB compromise after MV stent crossover implantation (21). Two sequential JB-POT procedures were performed sequentially on the LAD-D2 and LAD-S1 lesions, as shown in Figure 7. A third stent was then deployed in the proximal LAD-LM with a wire jailed in the LCX because the LCX was thought to be safe during stent crossover. Postprocedure IVUS examination showed that the stents had quite satisfactory apposition and expansion after JB-POT interventions. The struts just proximal to the branching point also showed good apposition and expansion, although they were avoided by re-POT (Figure 8). The whole procedure protected 3 SBs (1 diagonal branch, 1 septal branch and 1 LCX) by the same guide wire, costing only 40 min, 100 ml of contrast media and 350 mGry X ray radiation. No rewiring and kissing were required.

## Discussion

Repeated jailed balloon and rewiring SB are often troublesome in coronary stent implantation on bifurcation lesions with multiple risked SBs. The JB-POT protocol removes the rewiring maneuver and simplifies JB-PS to 3 main steps: jailed balloon stent implantation, jailed balloon POT and final re-POT. The major findings of this study were as follows: (a) JB-POT does not lead to imperfect stent implantation, as indexed by stent apposition and expansion in the bench test and clinical observation; (b) the success rate of JB-POT is quite satisfactory, as shown by the low endpoint event rate; and (c) JB-POT saves time, contrast media and radiation dosage and is easy to perform, especially for those who are just starting their career in coronary intervention.

The mechanism of SB complications during bifurcation stenting includes atheromatous plaque shift from MV to SB, carina shift toward the SB lumen, the presence of stent struts covering the SB ostium, coronary vasospasm, and the formation of a local thrombus covering the SB ostium. Based on the results from earlier studies, plaque shift and carina shift are considered to play a major role in this complication. Further studies also indicate that a shift of carina, but not of atheromatous plaque, was the main cause for SB narrowing. Previously, several studies were designed to explore risk factors for SB complications in crossover stenting protocols. Many risk factors, such as a small bifurcation angle, carina tip-branching point length and stenosis at the SB ostium, were associated with a significantly higher incidence of SB ostial stenosis (23–25). However, the overwhelming workload of clinicians requires a simple way to predict SB complications. Vassilev et al. and Longobardo et al. proposed a simple model to predict SB compromise called carina mismatch by QCA (18, 21). Unless the carina length was less than the SB ostium diameter, the SB would be liable to be blocked by carina or plaque shifts during MV stent implantation, similar to the relationship between a door and a doorframe. Because many factors, such as bifurcation angle and stenosis at SB ostium, influence the relationship between carina length and SB ostium diameter, the carina mismatch model is quite a comprehensive and easy-to-grasp model to predict SB compromise during MV stenting. We found that this model was quite applicable in real-world clinical practice not only in true bifurcation lesion (Medina 0,1,1; 1,0,1; 1,1,1) but also in false bifurcation lesion (Medina 0,1,0; 1,0,0 or 1,1,0) interventions. Therefore, the current study adopted the carina mismatch model to predict whether the SB was endangered and needed jailed balloon protection.

A few strategies have been proposed to protect an endangered SB from closure during MV stent crossover implantation, namely, predilation of the SB, the jailed wire technique and the JBT. Predilation of the SB was applied to prevent SB compromise but was shown to have limited effects on SB protection. The main reason is that predilation cannot prevent stent-induced carina shift, so EBC expert consensus recommends predilation only when the SB ostium is severely stenotic (26). Another constantly used SB protective measure was the jailed wire technique, which also has limited effects on SB complication prevention. Unlike the unsatisfactory protective effects of the former 2 strategies, the JBT was proven to be the most effective strategy in the prevention of SB compromise (3, 9, 10). In addition, provisional stenting was recommended for most bifurcation lesion interventions. Therefore, JB-PS became a useful strategy in treating bifurcation lesions with endangered SBs in certain clinical scenarios. The traditional JB-PS protocol includes stent deployment with a jailed balloon in the SB, rewiring the SB, proximal optimization, post-dilation of the stent, SB ostium dilation, the kissing balloon technique, and rePOT, which greatly increase contrast medium use and procedure duration times. Saito et al. presented a modified jailed balloon technique, which showed more satisfactory effects than conventional JBT (8). Theoretically, side branch was at risk if balloon for POT was not positioned accurately. When there were 2 or more risked SBs needing sequential protection, rewiring and other steps were supposed to be repeated. With repeated rewiring, device delivery might cause device entanglement, SB protection failure and even procedure failure, which sometimes are very troublesome for interventionists. Our modified JB-POT protocol includes only 3 steps: MV stent deployment with a jailed balloon in the SB, post-dilation and POT with a jailed balloon in the SB, and final distanced rePOT (POT balloon positioned 2 mm from the branching point). The cordial modification of this protocol prevents the most troublesome and time-consuming steps of the rewiring, cross-strut SB ostial dilation and final kissing in complicated bifurcation lesion treatment.

One concern about this simplified protocol is that retrieval of the jailed balloon might leave a “gap” (malapposition) between the struts and vessel wall. A previous study showed that OCT could conveniently be acquired from silicon bifurcation phantoms, and OCT had quite strong resolution power to recognize malapposition (27). Therefore, this silicon bifurcation phantom is suitable for testing the reliability of the simplified protocol. In this bench test model, we found that the stent struts rebounded and narrowed the “gap” after the jailed balloon was retrieved. However, slight malapposition sometimes existed after JB retrieval in the bench test if we pulled the jailed balloon with force. Therefore, jailed balloon retrieval might leave a “gap” when there is a calcification lesion underneath the stent under clinical circumstances. For the best safety in clinical observation, we added a distanced re-POT in the final design to eliminate the gap as best we could. Distanced re-POT was realized by positioning the POT balloon 2 mm away from the SB branching point to avoid POT dilation-induced carina shift and subsequent SB compromise. As a result, the simplified JB-POT protocol did not induce significant differences in the malapposition rate or expansion rate in the present bench study. Another concern is whether the modified JB-POT could effectively prevent SB compromise. If the SB blood flow was blocked or could hardly be restored, it was defined as a JB-POT failure event (primary endpoint event). There were no such events occurring in the present observation. If SB ostium stenosis was aggravated and could be easily repaired, it was defined as a secondary endpoint event. Under clinical circumstances, most SB compromises occur in LAD-D bifurcation lesion interventions. The SBs of Medina (1,1,1) bifurcation lesions are mostly vulnerable to SB closure during MV single-stent crossover interventions. There was 1 such event in our observation, which is acceptable for bifurcation intervention. According to previous studies, the incidence of SB complications was 5–15% using jailed wire-based provisional stenting in bifurcation interventions. The application of the JBT could decrease the incidence to below 5% (23). In the present study, we did not find any non-rescuable SB closure, suggesting that the JB-POT protocol is an effective strategy for the protection of risked SBs in provisional stenting.

It might be not safe for severe calcified lesions because jailed balloon might be difficult to retrieve after POT and post-dilation. However, we think it would be safer if we correctly utilized atherosclerotic plaque debulking techniques like rotational atherectomy or excimer laser coronary angioplasty (ELCA). In respect to in-stent lesions, the sandwich formed by two layers of metal struts and jailed balloon might cause detrimental results. Therefore, we do not think it appropriate to routinely use JB-POT under such circumstances.

## Conclusions

The JB-POT protocol, which tremendously simplifies the current standard provisional stenting procedure in complicated bifurcation lesion treatment, shows acceptability in safety and efficacy. Hence, it might help save time, reduce contrast consumption and reduce complications in the intervention of high-risk bifurcation lesions, especially those with multiple risked SBs.

### Limitations

First, we were not able to accurately compare JB-POT with the standard JB-PS protocol because the present study was not a prospective randomized controlled trial. Second, the patient count was limited to those with SB complications during JB-POT, and a multicenter RCT is anticipated to obtain a more reliable conclusion. Third, we used one brand of phantom to allow a reliable comparison in the bench test. Stent selection is critically important when performing JB-POT since marked differences exist between stent platforms, including stent strut thickness, elongation capability and the number of connections between struts in the real world. We chose one stent known for its platform performance in terms of crossability, stent strut thickness, deformation capability and early endothelial coverage in animal models. For this reason, we could not include any comparison with other stent platforms in the present study. Finally, we have not acquired angiographically follow-up results and the MACE data of long term follow-up. Further observation was still needed to determine the efficacy and safety of this protocol.

## Data Availability Statement

The raw data supporting the conclusions of this article will be made available by the authors, without undue reservation.

## Ethics Statement

The studies involving human participants were reviewed and approved by the Ethics Committee of Tangdu Hospital. The patients/participants provided their written informed consent to participate in this study.

## Author Contributions

WG proposed this strategy and applied it clinically from December 2018. DL conducted the bench test. WM and HL analyzed the data. PL and BB assisted WG to carry out the operation. DL and MZ drafted this paper. All authors contributed to the article and approved the submitted version.

## Funding

This work was supported by Tangdu Innovative Development Project (2021LCYJY044).

## Conflict of Interest

The authors declare that the research was conducted in the absence of any commercial or financial relationships that could be construed as a potential conflict of interest.

## Publisher's Note

All claims expressed in this article are solely those of the authors and do not necessarily represent those of their affiliated organizations, or those of the publisher, the editors and the reviewers. Any product that may be evaluated in this article, or claim that may be made by its manufacturer, is not guaranteed or endorsed by the publisher.

## Abbreviations

SB: side branch
JB-POT: jailed balloon proximal optimization technique
CBLs: coronary bifurcation lesions
MV: main vessel
OCT: optical coherence tomography
MSA: minimum stent area
JB-PS: jailed balloon-based provisional stenting
POT: proximal optimization technique
JBT: jailed balloon technique
Re-POT: repeated POT
EBC: European Bifurcation Club
TIMI: thrombolysis in myocardial infarction
LM: left main coronary artery
QCA: quantitative coronary angiography
IVUS: intravascular ultrasound
MACE: major adverse cardiovascular events
TLR: target lesion revascularization
MI: myocardial infarction
STEMI: ST segment elevation myocardial infarction
NSTEMI: non-ST segment elevation myocardial infarction
LAD: left anterior descending coronary artery
LCX: left circumflex coronary artery
D1: the first diagonal branch
D2: the second diagonal branch
OM: obtuse marginal branch
ELCA: excimer laser coronary angioplasty.
